# Genomic Analysis for the Safety Assessment of a Potential Probiotic Strain *Pediococcus pentosaceus* BBS1 Isolated From Lao Fermented Bamboo Shoots (*Nor Mai Som*)

**DOI:** 10.1002/mbo3.70048

**Published:** 2025-09-09

**Authors:** Viengvilaiphone Botthoulath, Ida F. Dalmacio, Francisco B. Elegado, Lawrence Yves Uy, Hsiang‐Chun Lin

**Affiliations:** ^1^ Graduate School University of the Philippines Los Baños (UPLB) Laguna Philippines; ^2^ Microbiology Division Institute of Biological Sciences, UPLB Laguna Philippines; ^3^ Biotechnology for Industry, Energy and the Environment Program National Institute of Molecular Biology and Biotechnology (BIOTECH), UPLB Laguna Philippines; ^4^ Department of Agronomy National Taiwan University Taipei Taiwan

**Keywords:** annotation, *Pediococcus pentosaceus* BBS1, safety assessment, whole‐genome sequencing (WGS) analysis

## Abstract

Currently, there is an increasing use of whole‐genome sequencing (WGS) studies to investigate the molecular taxonomy, metabolic properties, enzyme capabilities, and bioactive substances of lactic acid bacteria (LAB) species. In this study, the genome of strain *Pediococcus pentosaceus* BBS1 was sequenced using the Illumina HiSeq. 2500 platform to determine its classification, annotate its main features, and evaluate its safety characteristics. Results showed an average nucleotide identity (ANI) value of 99.60% for *Pediococcus pentosaceus* BBS1. *P. pentosaceus* BBS1 genome was composed of a 1,840,613 bp circular chromosome with a GC content of 37.23%, which contained 1778 predicted protein‐coding sequences (CDSs). Rapid Annotation using Subsystems Technology (RAST) linked to the Kyoto Encyclopedia of Genes and Genomes (KEGG) analysis revealed that strain BBS1 possesses l‐acetate dehydrogenase (*l‐LDH*; EC 1.1.1.27) and d‐lactate dehydrogenase (*d‐LDH*; EC 1.1.1.28), which are the genes responsible for lactic acid production. Additionally, it was found to contain *linamarase*, or *β‐glucosidases* (EC 3.2.1.21), a gene that functions for cyanide degradation. Significantly, the safety studies carried out using WGS confirmed the absence of virulence factors, biogenic amines, and antibiotic‐resistance genes in BBS1. Our previous research conducted in this study have shown that BBS1 possesses probiotic features, including tolerance to the simulated artificial gastrointestinal tract, bacterial adhesion, antibacterial activity, and antioxidant function. The findings provided herein significantly enhanced the known information on BBS1, supporting its potential application in promoting health through food products.

## Graphical abstract

*Pediococcus pentosaceus* BBS1 was analyzed by whole genome sequencing for its classification, annotated its main features for significant genes, and safety characteristics. The strain has the potential to be used as a beneficial probiotic starter culture, improving the quality of the fermented bamboo and related products.

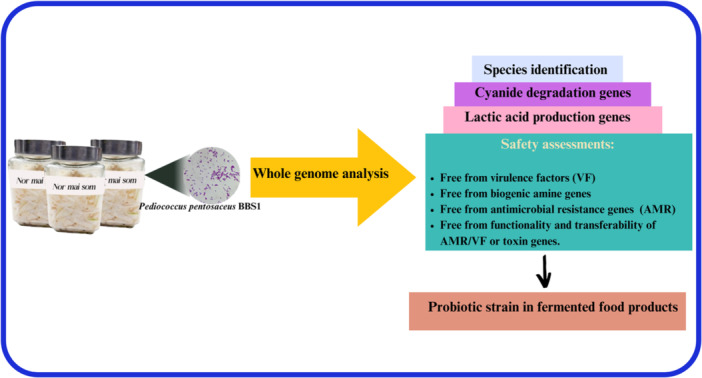

## 1 Introduction

Probiotic foods are a type of functional foods that are widely available worldwide and have reached a market value of US $46.55 billion in 2020 (Singh et al. 2018; Tarrah et al. 2020). Researchers have recently investigated the potential of several LAB as probiotics, including those from the genera *Bifidobacterium*, *Pediococcus*, *Lactococcus*, and *Enterococcus*. *Pediococcus pentosaceus* is classified in the family *Lactobacillaceae* within the order Lactobacillales (Zheng et al. 2020). Because of their ability to produce antimicrobial agents (acidophilucin A, lactacins A, pediocin, plantaricin, nisin Z, and reuterin, organic acids, hydrogen peroxide, and diacetyl, and due also to their probiotic properties (resistant to gastric acidity and bile salt, bacterial adhesion, antibacterial activity, susceptible to antibiotics, absence of biogenic amine production and virulence factors, and nonhaemolytic activity), they are recognized as non‐pathogenic microorganisms with a long history used as starter cultures in the food and beverage industry (Lv et al. 2014). Several studies have shown beneficial *P. pentosaceus* health effects like bioactivities such as improvement of lactose metabolism, anti‐inflammatory, anticancer, antioxidant, antibacterial, modulation of immune response, prevention of gastrointestinal infections, cholesterol‐lowering, and antihypertensive. This gives probiotic bacteria the potential to be used in biomedical and pharmaceutical applications (Jiang et al. 2020; Page and Pérez‐Díaz 2023; Rahman et al. 2024). *P. pentosaceus* has been widely exploited in food fermentation due to its safe traits and improvement of the attributes of fermented products in the food industry as a potential biocontrol approach against pathogenic microorganisms such as *Escherichia coli, Bacillus cereus*, *Staphylococcus aureus*, and *Listeria* spp., requires emphasis (Rahman et al. 2024). Jiang et al. (2021) reported that *P. pentosaceus* has been recently considered a trending alternative a future additive or probiotic candidate in food bio‐preservation because it can be applied as an antibacterial and anti‐virulence natural agent, its production of a wide range of antibacterial metabolites, and/or competitive exclusion mechanisms; thus, there is a need for producers and consumers to search for developing innovative food preservative approaches as “clean label” in varieties of food products. As a result, more research into *P. pentosaceus* is required for future applications. In the future, a thorough research of many *P. pentosaceus* strains should shed light on the benefits and disadvantages.

Whole‐genome sequencing (WGS) has resulted in a notable rise in the amount of sequencing‐based data used to analyze the systematics and molecular taxonomy, enzyme system, and bioactive chemicals of LAB species in great detail (Kwong et al. 2015). Several studies (Lv et al. 2014; Midha et al. 2012; Jiang et al. 2020; Page and Pérez‐Díaz 2023; Rahman et al. 2024) have examined the entire genome sequences of different *P. pentosaceus* strains. These investigations have enabled the introduction of novel methods to infer the evolutionary and divergent relationships among the strains. WGS is an effective technique for accurately analyzing and comprehending the genetic makeup of strains and the roles of LAB at the genomic level (Sharma et al. 2020). Furthermore, the safety of the starter cultures employed in the process of food fermentation has not consistently undergone a thorough assessment. The European Food Safety Authority (EFSA 2012) released guidelines for evaluating the safety of probiotics. These guidelines emphasize the importance of thoroughly examining the safety of live probiotic bacteria before using them in food products. The bacteria obtained from different sources must be free from any toxic substances and factors such as virulence factors, resistance genes, and biogenic amines. Additionally, they should be non‐pathogenic and unaffected by diseases such as infective endocarditis or digestive tract disorders (Gao et al. 2014; Dlamini et al. 2019). WGS should be included in the process of identifying strains and evaluating their safety, a practice that is becoming increasingly popular (Pariza et al. 2015). By utilizing WGS analysis, safety evaluations of bacterial strains can be carried out with significantly enhanced precision and thoroughness. Therefore, this study was conducted to: (1) sequence *P. pentosaceus* BBS1 strain, isolated from *Nor Mai Som*; (2) ascertain the classification and perform the annotation of the genome's main features; and (3) evaluate the safety characteristics (virulence factors, synthesis of biogenic amines, presence of antimicrobial resistance (AMR) genes, and bacterial mobile genetic elements) of strain via WGS, which will help facilitate the engineering of the strain for further use.

## 2 Materials and Methods

### 2.1 Bacterial Strains and Growth Media

*Pediococcus pentosaceus* BBS1 was isolated from Lao traditional fermented bamboo shoots. The strain was grown in MRS medium (HiMedia, India) for 18–24 h at 37°C and was maintained as stock cultures containing 30% glycerol at –80°C until further use. Before use in the different experiments, the isolate was sub‐cultured at least twice in MRS broth for 18–24 h at 37°C.

### 2.2 Whole Genome Sequencing Analysis

#### 2.2.1 Genomic DNA Extraction

Genomic DNA of BBS1 was extracted as described by Botthoulath et al. (2018), with slight modifications. Cells were prepared for DNA extraction when it was at the early exponential phase. The Quick‐DNA Fungal/Bacterial Miniprep kit (Zymo Research Corp, USA) was used following the manufacturer's instructions. After extraction, the bacterial DNA concentration and purity were assessed using a NanoDrop‐1000 spectrophotometer (Thermo Fisher Scientific, USA). The purity of the genomic DNA was checked with an OD260/OD280 ratio, which fell within the range of 1.8–2.0 (Chokesajjawatee et al. 2020). To assess the yield of genomic DNA, the mixture of the proportion of samples and 4X loading blue dye (3:1) was loaded into the gel electrophoresis using 1% (w/v) agarose containing *gelred* nucleic acid gel stain (Zymo Research Corp, USA). The gel was directly visualized under ultraviolet light to locate the clear bands, and the genomic DNA was submitted for WGS.

#### 2.2.2 Genome Sequencing and Genome Assembly

The genomic DNA of strain BBS1 was submitted for WGS using Illumina technology (Illumina Inc., Macrogen, Korea). The sequencing library was prepared using TruSeq kits and the library QC with the standard PacBio library protocol. Paired‐end reads with 100 bp setting was produced using a HiSeq. 2500 platform sequencing instrument. The sequence files, which were imported from BAM, SAM, or FastQ files, underwent evaluation using FastQC v0.11.7. This evaluation was conducted both before and after trimming to perform quality control checks on the raw sequence data obtained from high‐throughput sequencing approaches (Andrews 2010). Trimmomatic v0.3833 was used to trim reads (including adapter removal) and reject sequences having a pair base sequence quality score < 30 (Bolger et al. 2014). Next, the sequencing reads were utilized for the de novo assembly using Unicycler v0.4.7 (Wick et al. 2017) and also using Patric v3.6.9 (Davis et al. 2020), available at https://www.patricbrc.org/ with the default parameter set.

#### 2.2.3 Gene Prediction and Functional Annotation

Gene prediction and computation of annotation of protein‐coding genes were performed to determine the genomic features of strain BBS1 using Rapid Annotation using Subsystems Technology (RAST) (Aziz et al. 2008), available at https://rast.nmpdr.org/ with the default parameter set. A circular graphical genome map of this strain was created using Pathosystems Resource Integration Center (PATRIC) tool—Bacterial and Viral Bioinformatics Resource Center (BV‐BRC 3.29.20) (Brettin et al. 2015), available at https://www.bv-brc.org/.

#### 2.2.4 Identification of Species

Species identification of strain BBS1 was conducted by using both the 16S rRNA gene and the ANI (Chokesajjawatee et al. 2020). The 16S rRNA gene sequence was extracted from whole‐genome data and examined for contamination to establish the strain's species identification using a web‐based tool ContEst16S (Lee et al. 2017) available at https://www.ezbiocloud.net/tools/contest16s. The selected genome also undergoes ANI analysis for species identification using Orthologous Average Nucleotide Identity Tool (OAT) (Lee et al. 2016). The ANI value of 95%–96% was used as a standard for confirming the species of the strain (Richter and Rosselló‐Móra 2009).

### 2.3 Bioinformatic Analysis of Safety Assessments of *Pediococcus pentosaceus* BBS1

#### 2.3.1 Detection of Virulence Factors, Biogenic Amine, and Antimicrobial Resistance (AMR) Genes via Whole Genome Analysis

To discover potential virulence genes, a rigorous search was conducted utilizing specific criteria, including cut‐off values of > 80% identity and > 60% coverage. The virulence factor database (VFDB) (Liu et al. 2019) available at http://www.mgc.ac.cn/cgi-bin/VFs/v5/main.cgi was used to search for the presence of the virulence factors and toxin genes in the genome of strain BBS1. OriTfinder (Li et al. 2018), available at https://bioinfo-mml.sjtu.edu.cn/oriTfinder/ and the RAST linked to Kyoto Encyclopedia of Genes and Genomes (KEGG) database (Overbeek et al. 2014), available at https://rast.nmpdr.org/rast.cgi, were used as additional tools in determining the virulence factors and undesirable genes as recommended by EFSA Panel on Biological Hazards BIOHAZ (2011).

Biogenic amine genes, including arginine, cadaverine, histamine, ornithine, putrescine, spermidine, tyramine, and tryptamine in the BBS1's genome was searched using RAST through the KEGG pathways available at https://rast.nmpdr.org/rast.cgi.

The detection of the AMR genes in the genome of the BBS1 was done using three publicly available databases: ResFinder 4.1 (Zankari et al. 2017) available at https://cge.cbs.dtu.dk/services/ResFinder/, Comprehensive Antibiotic Resistance Database (Alcock et al. 2023) (CARD, RGI 5.0.0, CARD 3.0.3) available at https://card.mcmaster.ca, and also by the RAST through the KEGG pathways.

The possibility of transfer of the AMR gene, which is located in mobile elements such as conjugative plasmids, plasmids, and intact prophages, was also investigated. The presence of prophages in the genome was predicted using PHAge Search Tool Enhanced Release (PHASTER) tool available at http://phaster.ca/ (Prophage/Virus DB) (Arndt et al. 2016). The web‐based tool oriTfinder (Li et al. 2018), available at https://bioinfo-mml.sjtu.edu.cn/oriTfinder/ (database version: 1.1) was used to detect the Origin of Transfer (*oriT*), for the possibility of self‐transmission through conjugative plasmids.

## 3 Results

### 3.1 *Pediococcus pentosaceus* BBS1 Whole Genome Sequencing and Its Main Genomic Features

**Figure 1:**
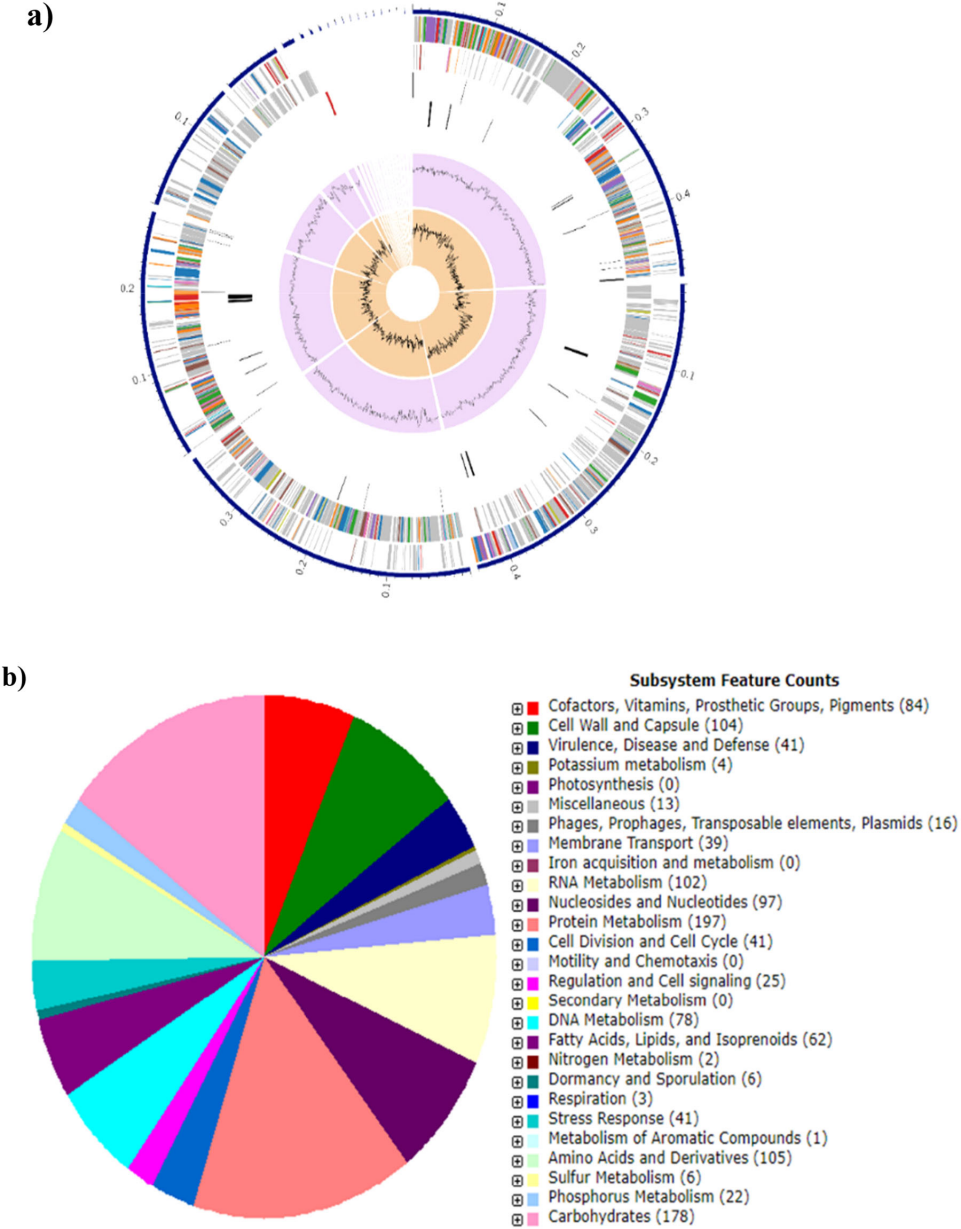
A circular graphical genome map of *P. pentosaceus* BBS1 analyzed by the PATRIC tool‐Bacterial and BV‐BRC 3.29.20. The contigs, CDS on the forward and reverse strands, RNA genes, CDS with homology to known virulence factors and antimicrobial resistance genes, GC content, and GC skew are listed from outer to inner rings (a). A description of the RAST‐linked annotation and related subsystems for this gene. The subsystem to which these genes belong is shown by the colors of the CDS on the forward and reverse strands (b).

The complete genome sequence for strain BBS1 was obtained through the Illumina HiSeq. 2500 platform. The major features of strain BBS1 genome comprising of 1,840,613 bp circular chromosome with a GC content of 37.23%. A total of 1778 protein‐coding sequences (CDSs) were identified as putative functions by the RAST server through gene prediction and annotation. These putative functions were spread throughout 286 subsystems. There were predicted to be 21 contigs, 54 *tRNA* genes, and three *rRNA* genes (Table 1). Using Patric v3.6.9, the circular graphical genome map was created, showing GC content, GC skew, and CDS on the forward and reverse strands (Figure 1a), which shows a count of the parts of a subsystem that are usually responsible for a certain biological process or structural complex (Figure 1b). The protein metabolism value of 197 made up most of this subsystem count, followed by the carbohydrate metabolism value of 178. This indicates that this strain has a significant capacity for protein metabolism.

**Table 1:** Genome features of *P. pentosaceus* BBS1 analyzed by RAST.

Attributes	Values
Genome size (bp)	1,840,613
GC content (%)	37.23
Contig N50	288,535
Contig L50	3
Number of contig	21
Number of protein‐coding sequences (CDSs)	1778
Number of subsystems	286
*tRNA*	54
*rRNA*	3

### 3.2 Identification of Species by Whole‐Genome Sequencing (WGS)

**Figure 2:**
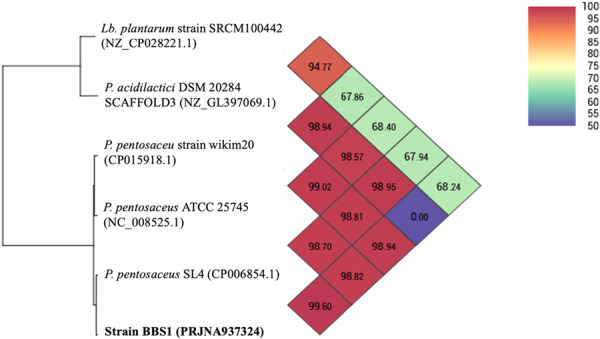
Identification of strain BBS1 using the OAT. The OrthoANI calculation of strain BBS1 revealed a high similarity ANI value of 99.60% with *P. pentosaceus* SL4 and *P. pentosaceus* ATCC 25745 (98.70%).

Strain BBS1 was previously identified as *P. pentosaceus* based on its 16S rRNA gene sequence. The WGS analysis confirmed that the strain BBS1 refers to the species *P. pentosaceus* (BioProject accession number is PRJNA937324) (Figure 2). According to the ANI analysis, strain BBS1 has the highest similarity to *P. pentosaceus* SL4 (CP006854.1) with an ANI value of 99.60%.

### 3.3 Lactic Acid Production

As indicated in Table 2, analysis using the RAST tool linked to the KEGG pathway revealed l‐lactate dehydrogenase (l‐LDH; EC 1.1.1.27) and d‐lactate dehydrogenase (d‐LDH; EC 1.1.1.28) as the genes responsible for lactic acid production in strain BBS1 genome, which was compared with *P. pentosaceus* ATCC 25745 genome as a reference strain. It is evident that the *ldhA*, d‐lactate dehydrogenase (EC 1.1.1.28) gene sequence was present in only one position with a length of 996 bp in strain BBS1 genome. For the *ldh*, l‐lactate dehydrogenase (EC 1.1.1.27) was detected at two positions in strain BBS1 with lengths of 963 and 921 bp.

**Table 2:** List of the d‐lactate and l‐lactate formation of *P. pentosaceus* BBS1 analyzed by using RAST linked to KEGG pathway.

Feature ID	Gene ID	Type	Coordinates	Strand	Length (bp)	Function
**d‐lactate formation**						
fig|1255.508.peg.1037	K03778	CDS	Start: 328307 Stop: 327312	—	996	ldhA; d‐lactate dehydrogenase (EC 1.1.1.28)
**l‐lactate formation**						
fig|1255.508.peg.697	K00016	CDS	Start: 425636 Stop: 424674	—	963	LDH, ldh; l‐lactate dehydrogenase (EC 1.1.1.27)
fig|1255.508.peg.1661	K00016	CDS	Start: 18569 Stop: 19489	+	921	LDH, ldh; l‐lactate dehydrogenase (EC 1.1.1.27)

### 3.4 Linamarase

**Figure 3:**
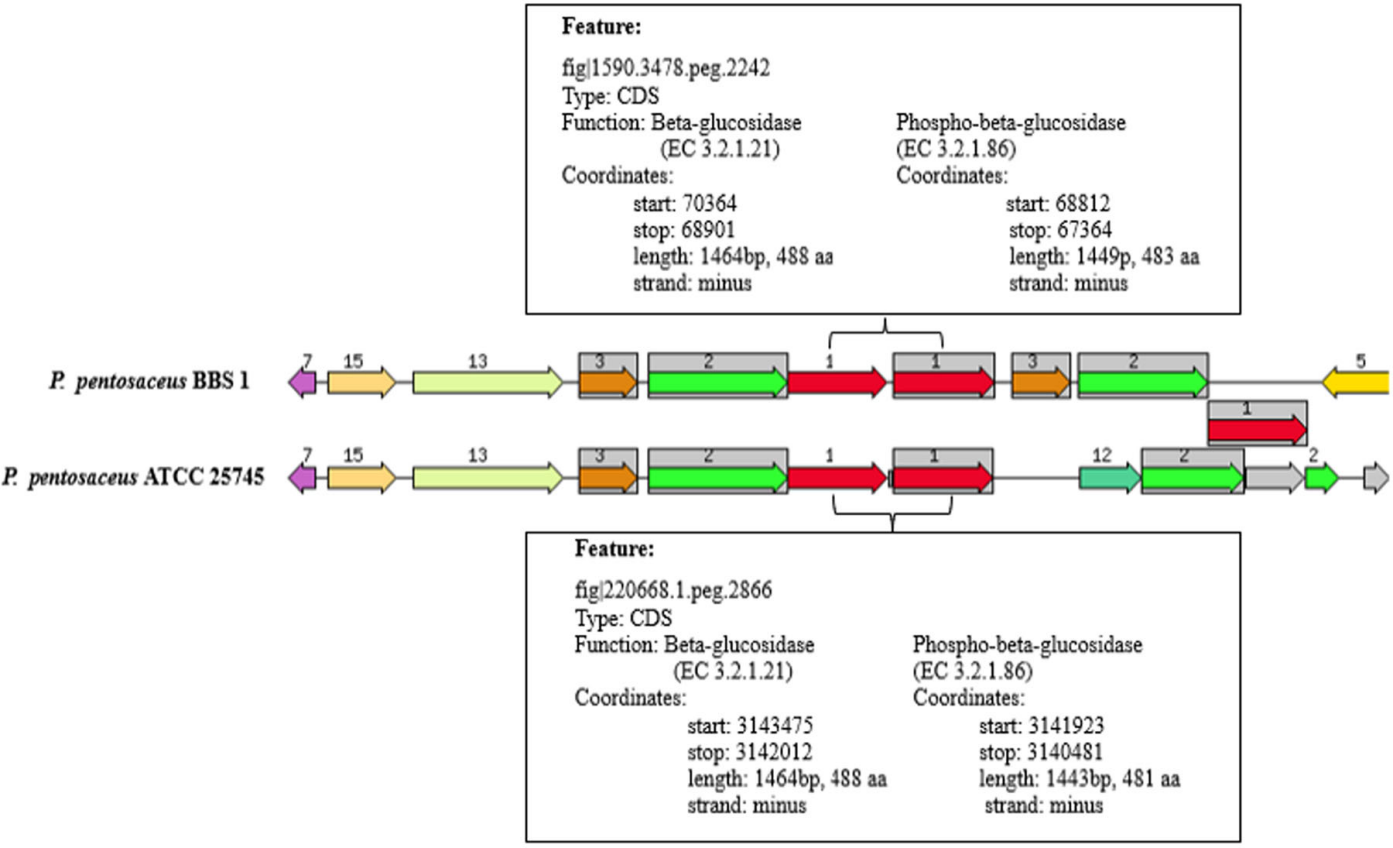
Comparison of the chromosomal gene regions for β‐glucosidase (EC 3.2.1.21) of *P. pentosaceus* BBS1 with other reference strains, analyzed by RAST linked to Kyoto KEGG pathway. The graphic is centered on the focus gene, which is red and numbered 1 (included in gray background boxes), genes that probably share other functional features.

The existence of linamarase genes in the strain's genome was analyzed using the RAST program linked to the KEGG pathway. As shown in Figure 3, the genomic property of BBS1 was compared with *P. pentosaceus* ATCC 25745 genome as a reference strain. BBS1 exhibited the same centered focus genes of β‐glucosidase (EC 3.2.1.21) with the length of DNA sequence at 1464 bp and 488 aa, as well as 6‐phospho‐β‐glucosidase (EC:3.2.1.86) (1449 bp and 483 aa), which are depicted in red and numbered 1 (included in gray background boxes), indicating that they likely share functional characteristics. This can be explained by the fact that strain BBS1 is uncertain of the precise function of these enzyme features, but one of them is most likely active.

### 3.5 Whole Genome Analysis on the Safety Concerns in *P. pentosaceus* BBS1

#### 3.5.1 Detection of Virulence Factor Genes

When virulence factor genes were searched for in *P. pentosaceus* BBS1 using the VFDB, no virulence gene sequences matched the cut‐off values of > 80% identity and > 60% coverage (Table 3). The OriTfinder analysis was used to determine additional virulence factors. Results showed that strain BBS1 was found to contain *ClpP*, *has* (*A, B*, and *C*), and *bsh* genes, which encode for stress survival, immune modulation, and bile salt hydrolysis, respectively. Similar patterns in the genes and proteins related to the *bsh* gene were found using RAST analysis, which were linked to KEGG by “Brite.” However, the gene encoding for Hemolysin III family, *YqfA*, was not found.

**Table 3:** Detection of virulence factor genes analyzed by VFDB and by RAST linked to KEGG pathway in *P. pentosaceus* BBS1.

BBS1				
Virulence factor category	Gene ID/Accession number	Related gene	Product/function	Result
**Under virulence factor database (VFDB)** Not found				
**Under OriTfinder**				
Stress survival	lmo2468 NP_465991	*ClpP*	– ATP‐dependent *Clp* protease proteolytic subunit – Serine protease involves proteolytic enzyme that is necessary for growth under stressful conditions (Gaillot et al. 2001)	Found
Immune modulation; Antiphagocytosis	SPY_RS09095 WP_010922799	*hasA; hasB; hasC*	– UTP‐‐glucose‐1‐phosphate uridylyltransferase HasC – GAS capsular hyaluronate chemically resembles human connective tissue. Consequently, the capsule discourages C3b binding and makes the bacteria appear “self” to the immune system, preventing phagocytosis (Ashbaugh et al. 1998)	Found
Bile salt hydrolysis/stress survival	lmo2067 NP_465591	*bsh*	– Bile salt hydrolase – Important for intestinal persistence of *L. monocytogenes*; involved in overcoming the acute toxicity of bile and bile salts (Begley et al. 2005)	Found
**Under RAST linked to KEGG via “Brite” Genes and Proteins**				
Bile hydrolysis	K01442	*bsh*	– Choloylglycine hydrolase (EC 3.5.1.24) – Bile salt hydrolysis is a crucial step in fat metabolism (Ren et al. 2018)	Found
Hemolysin III family	K11068	*YqfA*	Predicted membrane channel‐forming protein YqfA	Not found

#### 3.5.2 Detection of Biogenic Amine Genes

Strain BBS1 genome did not contain any biogenic amine genes, including those for arginine, cadaverine, histamine, ornithine, putrescine, spermidine, spermine, tyramine, and tryptamine, as determined by the RAST analysis via KEGG pathways (Table 4).

**Table 4:** Detection of biogenic amine genes analyzed by RAST linked to KEGG pathway in *P. pentosaceus* BBS1.

BBS1				
Name	Gene ID	Enzyme name	Product	Result
Biogenic amine formation via arginine and proline metabolism				
	K01583 K01584 K01585 K02626	Arginine decarboxylase [EC:4.1.1.19]	Arginine→agmatine	Not found
	K01476	Arginase [EC:3.5.3.1]	Arginine→ornithine	Not found
	K01480	Agmatinase [EC:3.5.3.11]	Arginine→putrescine	Not found
	K00797	Spermidine synthase [EC:2.5.1.16]	Putrescine→spermidine→spermine	Not found
	K01581	Ornithine decarboxylase [EC:4.1.1.17]	Ornithine→putrescine	Not found
Histidine metabolism	K01590	Histidine decarboxylase [EC:4.1.1.22]	Histidine→histamine	Not found
Lysine degradation	K01582	Lysine decarboxylase [EC:4.1.1.18]	Cadaverine production	Not found
	K23385	d‐ornithine/d‐lysine decarboxylase [EC:4.1.1.116]	Cadaverine production	Not found
Tyrosine metabolism	K22329 K22330	Tyrosine decarboxylase [EC:4.1.1.25]	Tyrosine→tyramine	Not found
Tryptophan metabolism	K01593	Tryptophan decarboxylase [EC:4.1.1.28]	Tryptophan→tryptamine	Not found

#### 3.5.3 Detection of Antimicrobial Resistance Genes (AMR)

The distribution of AMR genes in strain BBS1 was analyzed bioinformatically based on three databases: ResFinder 4.1, RAST through the KEGG pathways, and CARD (RGI 6.0.1). Table 5 demonstrated that no AMR genes were found in the genome of this strain using ResFinder 4.1's default settings, which were 90% threshold and 60% minimum length. However, the RAST analysis of the KEGG pathways revealed β‐lactam resistance. Likewise, using CARD analysis, strain BBS1 exhibited low identities of 32.43% and 48.57% for vancomycin resistance (*vanT* gene in *vanG* cluster) and quaternary ammonium resistance, respectively.

**Table 5:** List of antimicrobial resistance genes analyzed by RAST linked to KEGG pathway and by CARD, RGI 5.0.0 tool in *P. pentosaceus* BBS1.

AMR genes detection				
Not found any AMR genes in BBS1 under ResFinder4.1 tool				
Under RAST through the KEGG pathways				
Resistance	Kegg ID	Gene name	Product/function	Output
β‐lactam resistance	K17836	β‐lactamase class A [EC:3.5.2.6]	Penicillin‐binding proteins (PBPs)	Found
**Under CARD, RGI 6.0.1 tool**				
**Resistance**	**AMR gene family**	**Drug class**	**Resistance mechanism**	**% Identity of matching region**
Vancomycin resistance (*vanT* gene in *vanG* cluster)	Glycopeptide resistance gene cluster, *vanT*	Glycopeptide antibiotic	Antibiotic target alteration	32.43%
Resistance to quaternary ammonium, qacJ	Small multidrug resistance (SMR) antibiotic efflux pump	Disinfecting agents and antiseptics	Antibiotic efflux	48.57%

#### 3.5.4 Bacterial Mobile Genetic Elements (MGEs)

Strain BBS1 was analyzed for plasmids using *oriT*finder tool (Table 6). None of this strain's plasmids had any *oriT* of a conjugative plasmid or a chromosome‐borne integrative and conjugative element. This indicates that self‐transmission through conjugative transfer is unlikely to occur. Three prophage regions have been found for the bacteriophages in strain BBS1, all of which are found in chromosomes and are comprised of one intact region and two incomplete sections, according to the PHASTER tool. No AMR/virulence factor (VF) or toxic genes were located within the prophage regions.

**Table 6:** Summary of the possibility of gene transfer in *P. pentosaceus* BBS1 genomes analyzed by Origin of Transfer (*oriT*), and PHAge Search Tool Enhanced Release (PHASTER) tools.

Under *oriT*Finder tool (Plasmids)				
BB1	Conjugation	No *oriT* was predicted		
Under PHASTER tool (prophages)				
Region	**Prophage length**	**Completeness**	**Total number of proteins**	**Most Common Phage (number of gene hit)**
Chromosome BBS1				
1	14.4 kb	Incomplete	20	PHAGE_Brocho_BL3_NC_015254(2)
2	42.4 kb	Intact	60	PHAGE_Lactob_Sha1_NC_019489(15)
3	7.3 kb	Incomplete	13	PHAGE_Lactob_L_NC_047983(3)

## 4 Discussion

*P. pentosaceus* BBS1 exhibited the most potent inhibition against several indicator organisms. The previous test results also showed that it performed satisfactorily in its resistance to cyanide and its linamarase activity. Furthermore, it demonstrated survival or resistance to acidic conditions with a pH of 2.0 and a concentration of bile salts of 0.5% (Botthoulath et al. 2024; Botthoulath et al. 2025). Moreover, there were no identified safety issues related to antibiotic resistance or hemolytic activity (Botthoulath et al. 2025). Therefore, strain BBS1 had interesting traits that make it worthy for further more research. These include WGS, genomic approaches for assessing safety, and use as a starter culture in the fermentation of bamboo shoots.

Strain BBS1 was previously isolated from *Nor Mai Som*, a Lao traditional fermented bamboo shoots and was subjected to analysis by WGS. A comparative genomics investigation of *P. pentosaceus* strain IE‐3, reported that the number of coding genes involved in protein metabolism was the highest, which is similar with the annotation findings from this study (Midha et al. 2012). In addition, the primary functions of *P. pentosaceus* LI05 were focused on the putative functions of protein metabolism and carbohydrate metabolism, which provide molecular support for the strain to metabolize various amino acids and sugars (Lv et al. 2014). Protein and carbohydrate metabolisms serve as essential components for the proper functioning of a biological cell. As a result, the main energy sources for microorganisms' cell growth and development are proteins and carbohydrates. For example, LAB can acquire carbs from its surroundings and/or remove genes related to carbohydrates when none are needed (Yang et al. 2022). Moreover, strain BBS1 exhibited subsystem feature annotations of the cell wall and capsule of 104, indicating that this strain can form biofilms, thereby enhancing its resistance to externally hostile factors. The genomic data of strain BBS1 serves as a scientific foundation for its prospective application in fermented food products.

Identification of strain BBS1 was confirmed by WGS. The OrthoANI values of strain BBS1 was compared to other species, including *P. pentosaceus* ATCC 25745 (NC 008525.1) and *P. pentosaceu*s strain wikim20 (CP015918.1), which were above 98%. Similarly, *L. plantarum* strain SRCM100442 (NZ CP028221.1) and *P. acidilactici* DSM 20284 SCAFFOLD3 (NZ GL397069.1) were rooted as outgroups because their ANI values were significantly lower than the 95%–96% cut‐off threshold (Lee et al. 2016; Richter and Rosselló‐Móra 2009). Strain BBS1 demonstrated values of 0.01 against all *P. pentosaceus* strains when analyzed using the genome‐to‐genome distance calculator (GGDC). Corresponding with the findings of Jiang et al. (2020), all strains isolated various environments, such as human feces, belonged to *P. pentosaceus*, as evidenced by an ANI value of > 98%.

Microbial fermentation has traditionally produced lactic acid, which is widely used in several industries including food, pharmaceutical, cosmetic, and chemical. One such application is the production of bioplastics using the green polymer poly‐lactic acid (PLA) (Li et al. 2013). Microbial lactic acid production facilitated by enzymes is purer compared to chemical synthesis. The use of microbial fermentation is favored for the production of lactate because it allows for the utilization of only the pure l‐ and d‐lactic acid monomers, which are necessary precursors for PLA synthesis (Zheng et al. 2012; Ma et al. 2014). Chokesajjawatee et al. (2020) identified the two genes involved for the synthesis of d‐lactic acid such as lactate racemase (chr 00083) and d‐lactate dehydrogenase (chr_00684 and chr_1677), after examining the KEGG database. The optical purity of lactic acid generated by LAB is determined by the catalytic efficiency of the ldhL and ldhD encoded products (Zheng et al. 2012). In LAB, both *l‐LDH* and *d‐LDH* display diverse catalytic attributes and have significant roles in the lactic acid fermentation process. They are involved in the final step of the anaerobic glycolysis pathway, where they convert pyruvate and NADH into l‐lactic acid and d‐lactic acid, respectively (Arai et al. 2001; Sun et al. 2016). This current research has shown that the genes capable of producing lactic acid are present in strain BBS1, thereby, providing advantage when applied to food and other related products. Moreover, the existence of these genes can serve as a foundation for improving the efficiency of lactic acid production through genetic modification using plasmid transformation.

Strain BBS1 revealed linamarase activity based on the findings from the prior enzyme assay studies conducted in this study. Linamarase, also known as β‐glucosidase enzyme (EC 3.2.1.21), is frequently found in bacteria, particularly in LAB (Xie et al. 2022). The putative β‐glucosidase and phospho‐β‐glucosidase genes of LAB and their predicted organization in operons were analyzed by (Michlmayr and Kneifel 2014). Similarly, the β‐glucosidase in *L. paracasei* TK1501, which was isolated from naturally fermented congee, was analyzed using complete genome sequencing. The amino acid sequence of the β‐glucosidase in *L. paracasei* TK1501 was found to be 99.8% similar to that of *L. casei* ATCC334 (reference strain) (Xie et al. 2022). Generally, β‐glucosidases (EC 3.2.1.21) are often responsible for hydrolysing glycosidic linkages and removing glucopyranosyl residues from the nonreducing end of cellobiose, cellooligosaccharides, arylglucosides, and alkylglucosides. The substrate specificity, inducers, and cellular location of this enzyme are similar to those of different microbial enzymes (Michlmayr and Kneifel 2014; Lei et al. 1999; Fadahunsi et al. 2020). In the food industry, β‐glucosidases are used to liberate aromatic compounds from glucoside precursors, thereby enhancing the intended flavor and aroma in fruits and fermented products. The β‐glucosidase activities of various lactic acid bacteria (LAB), such as *L. plantarum, L. pentosus*, *L. brevis, P. pentosaceus, Leuconostoc*, and *Weissella*, have been used for eliminating the bitterness of linamarin, a toxic cyanogenic glucoside (Nout and Sarkar 1999; García‐Cano et al. 2020). Based on genetic data indicating the presence of β‐glucosidase genes corresponding to the previous test results for linamarase activity, strain BBS1 offers a valuable advantage to the food sector. Specifically, it can be used as a starter culture to decrease cyanide levels effectively. This could potentially facilitate the application of techniques for genetic engineering that utilize plasmid transformation to enhance the synthesis of linamarase in food fermentation processes.

Given the importance of safety, it is imperative to perform characterization of strains to identify any potentially undesirable features of all bacterial strains present in a probiotic product. WGS can also offer a detailed examination of genetic composition, including the possibility of horizontal gene transfer facilitated by plasmids, phages, integrons, and transposons (Peng et al. 2023).

The possibility of illnesses resulting from the presence of virulence‐encoding genes in specific genomic areas has drawn a lot of interest recently (Vesterlund et al. 2007). No virulence‐related genes were investigated in *P. pentosaceus* ST65ACC, a strain isolated from artisanal raw‐milk cheese (Oliveira et al. 2023). However, most of the genes were found to be defensive or unconventional virulence factors. This is similar to our investigation that the non‐offensive virulence factors identified in the current study, using other tools such as OriTfinder and RAST linked to the KEGG database, showed that no hemolysin genes were found in the BBS1 genome. This corresponds to our previous studies on hemolytic test using blood‐sheep agar, which did not exhibit hemolytic activity (Botthoulath et al. 2025). The genes were found as virulence factors in the virulence factor databases as they were associated with the adaptability, survival, or adhesion of pathogenic bacteria to their hostile or host environment. However, in the absence of other pathogenic mechanisms, these genes can be considered helpful to the bacterium as they enhance bacterial fitness and may be beneficial in situations when viable cells are needed, especially when employed as starter cultures, and also function as probiotics (Chokesajjawatee et al. 2020).

Biogenic amines (BA) cause concern in fermented food due to their toxic effects, ability to produce hypertension, and effects on the digestive system (Swetwiwathana and Visessanguan 2015). BA is largely produced by the synthesis of nitrogenous substances through the process of decarboxylation of free amino acids using decarboxylase, amination, and transamination of aldehydes and ketones. The corresponding BA is produced by removing the alpha carboxyl group from an amino acid (Özogul and Hamed 2018). For example, histidine is decarboxylated to produce histamine; tyrosine generates tyramine; lysine is decarboxylated to make cadaverine; and ornithine can be decarboxylated to generate putrescine; putrescine can be transformed into spermidine, which can then be used to produce spermine (Özogul and Hamed 2018; Landete et al. 2011]. Many BAs have been detected in *Enterococcus*, *Lactobacillus* and *Pediococcus* spp., which are major BA producers in fermented foods. Microbial strains intended for use in fermented food with enhanced microbial activity, may lead to undesired accumulation of BA. No genes are associated with the production of BA in LAB (Chokesajjawatee et al. 2020; Oliveira et al. 2023). Theoretically, the levels of BA present in food products are influenced by various parameters, such as the quality and quantity of microbiota, chemico‐physical variables, fermentation hygiene, precursor amino acids, pH, temperature, and others (Oliveira et al. 2023). A bacterial strain that does not have the genes required for BA synthesis, as suggested by EFSA, is referred to as a BA‐nonproducing strain and is considered to be non‐harmful. To evaluate the risk, it is essential to determine the actual production and accumulated levels of BA at the expected usage conditions, once the genes have been identified (Chokesajjawatee et al. 2020).

In the process of determining whether or not probiotic strains are safe to use, the phenotypic characteristics of those strains that are relevant to AMR are given significant consideration. Genomic analysis of microorganisms provides valuable data on AMR genes and their potential for transferability. This investigation is necessary to evaluate the potential risks associated with new probiotic strains (EFSA 2012). The studies by Chokesajjawatee et al. (2020) reported that there is an absence of occurrences of AMR genes in the genome of *L. plantarum* BCC9546 by utilizing the search function of ResFinder. However, CARD analysis identified 273 hits as AMR genes using a less stringent threshold, ranging from 19% to 61% identity and 16% to 30.7% coverage. Most of the hits were not AMR genes because the search criteria were not stringent enough. The research found that *L. plantarum* BCC954 was resistant to kanamycin sulfate based on the phenotype test. This correlates with this current study in which kanamycin resistance was detected in strain BBS1 by a previous phenotypic test, but no such antibiotic was found in its genome. This can be explained by the discovery in the genome of several genes linked to efflux pumps that cause multidrug resistance. It's probable that the strain's kanamycin resistance is a result of these efflux pumps. There are many factors, including the level of gene expression and the selectivity of the produced product towards its substrate. Similarly, the strain was sensitive to ampicillin/penicillin even though β‐lactamase genes were present. Before conducting further research, it is not possible to exclude the possibility of resistance to other β‐lactam medicines. The reason for this is because β‐lactamase is a diverse group of enzymes that exhibit variances in their ability to bind to different substrates (Philippon et al. 2016). Moreover, the majority of LAB‐carried intrinsic resistance or natural resistance to some antibiotics, including vancomycin, was due to the presence of d‐lactate or d‐serine residue instead of d‐alanine residue. This alteration in residue composition often hinders the binding of vancomycin. This resistance is not transmissible and chromosomally encoded (Fraqueza. 2015; Yushchuk et al. 2020; Selim 2022). Moreover, the EFSA's technical recommendations have determined that *P. pentosaceus* strains do not require the use of vancomycin breakpoints (EFSA 2012). The limited repertoire of AMR genes present in the databases can result in a failure to detect AMR using the default, stringent option. As a result of the ResFinder, CARD, and RAST linked KEGG databases mostly concentrate on AMR determinants of non‐pathogenic bacteria's genes, such as LAB, which are typically rarely listed. Because of this, it is important to be aware of the difficulties associated with identifying AMR genes in non‐pathogenic bacteria, utilizing the most recent version of these databases. However, it has been noted in numerous studies that safety concerns like AMR, virulence, and the capacity to produce toxins differ depending on the strain, even within the same species.

The primary concern regarding AMR/VF, or toxin genes present in beneficial non‐pathogenic bacteria, is the possible transmission of these genes to other potentially harmful bacteria. This transfer could cause issues and reduce the treatment's overall efficacy. It is vital to research mobile genetic components like plasmids and bacteriophages to determine the risk involved. These elements are commonly employed for intercellular genetic exchange through transformation, conjugation and transduction processes (Li et al. 2018). Even if LAB, particularly *Lactobacillus* spp., were present in these genes, it may have minimal transferability of AMR/VF or toxin genes via MGEs (Kwon et al. 2021).

Based on the findings of the present study, the use of numerous tools for the search was accurate and effective for its intended purpose. Thus, strain BBS1 does not pose a safety risk with regard to its functionality and transferability of AMR/VF or toxin genes. The aforementioned discoveries present the possibility of utilizing BBS1 as a beneficial starter culture in food products.

## 5 Conclusion

Numerous research have employed WGS to analyze the genome of LAB for different objectives. In this particular study, the chosen strain from the Lao fermented bamboo shoots was identified as *Pediococcus pentosaceus* BBS1. The genome exhibited several key characteristics, including genome size, GC content, number of CDSs, tRNA, and rRNA. The research performed using the RAST tool linked to the KEGG pathway revealed the presence of l‐acetate dehydrogenase (*l‐LDH*; EC 1.1.1.27) and d‐lactate dehydrogenase (*d‐LDH*; EC 1.1.1.28) genes in strain BBS1. These genes are responsible for the synthesis of lactic acid. Furthermore, its genome also contained linamarase, or β‐glucosidases (EC 3.2.1.21), which degrades cyanide. Significantly, this bacterium did not possess any virulence factors, biogenic amines, or antibiotic resistance genes. The genomic data of strain BBS1 can be utilized to guide genetic engineering approaches, with the aim of enhancing particular products such as lactic acid or linamarase production. Therefore, strain BBS1 can be confidently used as a probiotic strain in fermented food products.

## Author Contributions

**Viengvilaiphone Botthoulath:** conceptualization, writing – original draft, methodology, software, formal analysis, writing – review and editing, visualization, investigation, validation. **Ida F. Dalmacio:** writing – review and editing, resources. **Francisco B. Elegado:** writing – review and editing, supervision. **Lawrence Yves Uy:** formal analysis, genome assembly and annotation. **Hsiang‐Chun Lin:** providing critical guidance in the bioinformatic analysis. All authors reviewed the results and approved the final version of the manuscript.

## Ethics Statement

The authors have nothing to report.

## Conflicts of Interest

The authors declare no conflicts of interest.

## Data Availability

Data will be made available on request to the corresponding authors.
